# Canadian healthcare workers’ mental health and health behaviours during the COVID-19 pandemic: results from nine representative samples between April 2020 and February 2022

**DOI:** 10.17269/s41997-023-00807-z

**Published:** 2023-08-07

**Authors:** Vincent Gosselin Boucher, Brook L. Haight, Camille Léger, Frédérique Deslauriers, Simon L. Bacon, Kim L. Lavoie, Eli M. Puterman

**Affiliations:** 1 School of Kinesiology, University of British Columbia, Vancouver, BC Canada; 2 Department of Psychology, Université du Québec à Montréal, Montreal, QC Canada; 3 Montreal Behavioural Medicine Centre, Centre Intégré Universitaire de santé et services sociaux du Nord-de-l’Ile-de-Montréal (CIUSSS-NIM), Montreal, QC Canada; 4 Department of Health, Kinesiology and Applied Physiology, Concordia University, Montreal, QC Canada

**Keywords:** Healthcare workers, Mental health, COVID-19, Health behaviour, Travailleurs de la santé, santé mentale, COVID-19, comportement de santé

## Abstract

**Objective:**

In the context of COVID-19, Canadian healthcare workers (HCWs) worked long hours, both to respond to the pandemic and to compensate for colleagues who were not able to work due to infection and burnout. This may have had detrimental effects on HCWs’ mental health, as well as engagement in health-promoting behaviours. This study aimed to identify changes in mental health outcomes and health behaviours experienced by Canadian HCWs throughout the COVID-19 pandemic.

**Methods:**

Nine representative samples (*N*_total_ = 1615 HCWs) completed the iCARE survey using an online polling firm between April 2020 (Time 1) and February 2022 (Time 9). Participants were asked about the psychological effects of COVID-19 (e.g., feeling anxious) and about changes in their health behaviours (e.g., alcohol use, physical activity).

**Results:**

A majority of the HCWs identified as female (65%), were younger than 44 years old (66%), and had a university degree (55%). Female HCWs were more likely than male HCWs to report feeling anxious (OR = 2.68 [1.75, 4.12]), depressed (OR = 1.63 [1.02, 2.59]), and irritable (OR = 1.61 [1.08, 2.40]) throughout the first two years of the pandemic. Female HCWs were more likely than their male counterparts to report eating more unhealthy diets (OR = 1.54 [1.02, 2.31]). Significant differences were also revealed by age, education level, income, parental status, health status, and over time.

**Conclusion:**

Results demonstrate that the impacts of COVID-19 on HCWs’ mental health and health behaviours were significant, and varied by sociodemographic characteristics (e.g., sex, age, income).

**Supplementary Information:**

The online version contains supplementary material available at 10.17269/s41997-023-00807-z.

## Résumé

**Objectif:**

Dans le contexte de la COVID-19, les travailleurs de la santé canadiens ont travaillé de longues heures, à la fois pour répondre à la pandémie et pour compenser pour les collègues qui n’étaient pas en mesure de travailler en raison d’infection et d’épuisement professionnel. Cela a pu générer un important impact sur leur santé mentale, ainsi que sur leurs engagements dans des comportements favorables à la santé.

**Méthodes:**

Neuf échantillons représentatifs (N_total_ = 1 615 travailleurs de la santé) ont répondu à l’enquête iCARE par l’intermédiaire d’une compagnie de sondage en ligne entre avril 2020 (temps 1) et février 2022 (temps 9). Les participants ont été interrogés sur les effets psychologiques de la COVID-19 (p.ex., le sentiment d’anxiété) et sur les changements dans leurs comportements de santé (p.ex., la consommation d’alcool, l’activité physique).

**Résultats:**

La majorité des travailleurs de santé répondants sont des femmes (65 %), ont moins de 44 ans (66 %) et ont un diplôme universitaire (55 %). Les femmes travailleuses de la santé étaient plus susceptibles de se sentir anxieuses (OR = 2,68 [1,75, 4,12]), déprimées (OR = 1,63 [1,02, 2,59) et irritables (OR = 1,61 [1,08, 2,40]) que les hommes au cours des deux premières années de la pandémie. Les femmes travailleuses de la santé étaient plus susceptibles d’avoir une alimentation plus malsaine (OR = 1,54 [1,02, 2,31]) que leurs homologues masculins. Des différences significatives ont également été révélées selon l’âge, le niveau d’éducation, le revenu, le statut parental, l’état de santé et à travers le temps.

**Conclusion:**

Les résultats démontrent que les impacts de COVID-19 sur la santé mentale et les comportements de santé des travailleurs de santé sont significatifs, mais varient selon les caractéristiques sociodémographiques (p.ex., le sexe, l’âge, le revenu).

## Introduction

Healthcare workers (HCWs) account for the largest sector of government employees in Canada (Statistics Canada, 2017). The Canadian Federation of Nurses Unions, which recruited 7358 Canadian nurses in 2019 just prior to the pandemic, reported that nurses have a high prevalence of depressive (36%), anxiety (26%), and panic (20%) disorders (Stelnicki & Carleton, 2021). They also tend to work longer shifts, more overtime, and more unpaid overtime, and have more conflict at work compared to those in the general Canadian population (Shields & Wilkins, 2005). Similarly, a 2017 National Physician Health Survey including over 2500 physicians reported they too experienced high levels of emotional exhaustion (26%), burnout (30%), depression (34%), and suicidal ideation (9-19%) even before the pandemic (Canadian Medical Association, 2018). Physicians have also been shown to experience more than twice as much high work stress (64% compared to 27%) as the general Canadian population (Statistics Canada, 2017; Wilkins, 2007). The mental health problems reported by HCWs appear to be a direct consequence of their workload, which is physically and emotionally demanding (Embriaco et al., 2007; Kirby, 2008; Martin, 2018).

### Psychological consequences of COVID-19 on HCWs

During the SARS-CoV-2 (COVID-19) pandemic, HCWs across the globe reported greater negative mental health consequences (e.g., stress, depression symptoms) compared to pre-pandemic levels (Bai et al., 2004; Marjanovic et al., 2007; Ménard et al., 2022). HCWs have, at different periods of the pandemic, experienced events that may have undermined their mental health (e.g., quarantine, or knowing someone who died from the virus; Spilg et al., 2022; Wu et al., 2020). Meta-analyses have examined the prevalence of different mental health outcomes in HCWs during the COVID-19 pandemic, and found a pooled prevalence of experiencing depressive symptoms ranging from 22% to 33%, anxiety symptoms ranging from 20% to 42%, post-traumatic symptoms ranging from 21% to 32%, and insomnia ranging from 39% to 42% (Aymerich et al., 2022; Batra et al., 2020; Johns et al., 2022; Li et al., 2021; Pappa et al., 2020). These global findings are consistent with those reported by Statistics Canada, whereby 33% of HCWs between November and December 2020 (n=18,000) reported overall poor mental health. In the report, more nurses (37%) and personal care workers (35%) reported overall poor mental health compared to physicians (27%; Statistics Canada, 2021).

Several studies have examined the mental health status of HCWs in Canada during the pandemic, but these were mainly assessed in 2020 exclusively and were restricted to depressive and anxiety symptoms (Binnie et al., 2021; Crowe et al., 2021; Havaei et al., 2021; Ménard et al., 2022). There has been little or no mention of changes in HCWs’ health behaviours (e.g., physical activity, recreational drug use) which may have further exacerbated their mental health decline during the pandemic (Carazo et al., 2022; Crowe et al., 2022; Robillard et al., 2021; Wilbiks et al., 2021). Additionally, while research in the general population demonstrates different impacts of the pandemic on mental health by sociodemographic factors such as gender, sex, minority status, and socioeconomic status, it remains uninvestigated in HCWs (Ettman et al., 2020; Pierce et al., 2020).

The aims of the current study were to (1) report mental health and health behaviour outcomes among Canadian HCWs during the first 21 months of the pandemic (9 cross-sectional population-based samples between April 2020 and February 2022), and (2) examine whether there were any differences in mental health and health behaviour outcomes as a function of sex, or other sociodemographic and health variables. By examining such differences, Canadian healthcare institutions may have new, important information to design programs to better support HCWs and optimize the physical and mental health of those most impacted by the pandemic.

## Methods

### Study design and recruitment

We report data from the International COVID-19 Awareness and Responses Evaluation (iCARE) Study (www.icarestudy.com) using 9 waves of the Canadian representative sample from April 2020 to February 2022 (see Table 1). Each wave included a new independent sample. The details and methodological background of the iCARE study have been published elsewhere (Bacon et al., 2021). Briefly, 9 cross-sectional age, sex, and province-weighted population-based samples of adults 18 years and older were recruited to complete online surveys by the Leger Opinion polling firm, which recruits participants through their closed, proprietary online panel. This panel of participants included over 400,000 Canadians, the majority of whom (61%) were recruited within the past 10 years. Two thirds of the panel were recruited randomly by telephone, with the remainder recruited via publicity and social media. Respondents were invited to complete the survey by email and did so voluntarily. Leger Opinion sent panelists a unique link to complete each survey so that they could not complete a survey more than once. Across the 9 assessments, 183,358 participants were invited to respond to the surveys (mean per survey = 20,373±4758 invitations), with a response rate of 15.9% (±4.3%, ranging from 11.4% to 25.0%).

**Table 1:** The 9 time periods and sample sizes of the Canadian representative sample from April 20, 2020 to February 2, 2022

Time	Total # of HCWs from each survey (*n*, %)	Total # of participants from each survey (*n*, %)
1: April 9 – April 20, 2020	176 (10.90)	3003 (11.11)
2: June 4 – June 17, 2020	162 (10.03)	3005 (11.12)
3: October 29 – November 11, 2020	163 (10.09)	3005 (11.12)
4: January 27 – February 7, 2021	164 (10.15)	3000 (11.10)
5: March 11 – March 23, 2021	178 (11.02)	3006 (11.12)
6: May 31 – June 14, 2021	220 (13.62)	3005 (11.12)
7: September 10 – September 20, 2021	189 (11.70)	3004 (11.11)
8: November 15 – December 3, 2021	171 (10.59)	3002 (11.11)
9: January 20 – February 2, 2022	192 (11.89)	3001 (11.10)
Total	1615	27,031

Online consent was provided by participants prior to completing the survey. No personal identifying information was collected. Participants were offered nominal compensation through the polling firm (participants collect points that can be traded in for gift cards); no direct compensation was provided by the research team. The study was approved by the Research Ethics Committee at the Centre intégré universitaire de santé et de services sociaux du Nord-de-l’Île-de-Montréal (CIUSSS-NIM; REB#: 2020-2099/03-25-2020). The present paper is presented in line with the Checklist for Reporting Results of Internet E-Surveys (CHERRIES, see Supplementary Table 1; Eysenbach, 2004).

### iCARE Survey Questionnaire

For the current set of analyses, participants were included when they answered “Yes” to the question “Are you a healthcare worker?”. A detailed account of the survey questions is provided elsewhere (online: https://osf.io/nswcm/) and questions specific to this study are included in Supplementary Table 2. Each of the nine surveys included an average of 75 questions and took 15–20 mins to complete. Questions were presented in the same order across surveys, but the response set order was randomized for questions with multiple subitems to reduce bias. Some questions were conditionally displayed based on responses to previous items to reduce the number and complexity of the items. Completing all questions was mandatory to move forward, but many questions included the option “I don’t know/I prefer not to answer”. All details about the survey development have been published elsewhere (Bacon et al., 2021).

#### Outcomes

Mental health outcomes were measured with 4 different items (anxious, depressed, isolated, irritable) assessing participants’ emotional status over the past month. For each mental health outcome, we asked the participant “Please rate the extent to which COVID-19 has impacted the following aspects of your life over the last month”, with the following possible answers: To a Great Extent, Somewhat, Very Little, and Not at All. *Health behaviour* changes were measured with 6 different items (physical activity, healthy diet, drinking alcohol, smoking cigarettes, using e-cigarettes, and using recreational drugs) assessing whether engagement in these behaviours changed (doing the behaviour a lot more to doing it a lot less) from pre-pandemic levels. For each health behaviour, we asked the participant “In general, how have the following behaviours changed since the start of COVID-19?”, with the following possible answers: I do this a lot more, I do this more, I do this as much as before, I do this less, I do this a lot less, I don’t do this. The variables responses were merged and dichotomized for mental health outcomes (1 = to a great extent, 0 = all other choices) and health behaviour changes (1 = I do this a lot less or I do this less [health positive behaviour: physical activity, healthy diet], I do this much more or I do this more [health negative behaviour: drinking alcohol, smoking cigarettes, using e-cigarettes, using recreational drugs]; 0 = all other choices; see Supplementary Table 2).

#### Moderator variables

The following sociodemographic and health variables were used as moderators for the analyses: sex, age, education level, household income, parental status, and health condition status (having or not).

### Data analysis

Descriptive statistics for the outcome and moderator variables at each time point are presented in Table 2. Pearson chi-square tests were performed to assess differences in mental health (1= to a great extent, 0 = all other choices) and health behaviours (1 = I do this a lot less/more, I do this more/less; 0 = all other choices) for each moderator variable. All items that included ‘I don’t know/I prefer not to answer’ responses were considered missing values, and statistical analyses were based on complete case records. Multivariate logistic regressions were conducted using the SAS logistic regression procedure (PROC LOGISTIC) to assess the associations between either mental health outcomes or health behaviours (dependent variables) with a series of sociodemographic and health factors (independent variables) informed from the existing literature (i.e., sex, age, education level, household income, parental status, and health condition status; Lavoie et al., 2022; Leach et al., 2021). All statistical tests included the weighting variables as a covariate and were two-sided, and a *p*-value < 0.05 was considered statistically significant. The analyses were performed using SAS, version 9.4.

**Table 2:** Weighted prevalence of sociodemographics per time - N_w_ (%)

Time^a^	All	1	2	3	4	5	6	7	8	9	Chi-square *p*-value
HCWs	1615	176 (10.9)	162 (10.0)	163 (10.1)	164 (10.1)	178 (11.0)	220 (13.6)	189 (11.7)	171 (10.6)	192 (11.9)	
Sex											
Female	818 (66.0)	91.9 (55.9)	93.5 (62.1)	93.4 (67.2)	86.2 (74.0)	92.1 (73.3)	94.3 (59.9)	85.9 (64.1)	88.0 (72.4)	99.6 (74.4)	**0.002**
Male	421 (34.0)	74.8 (44.1)	57.6 (37.9)	45.6 (32.8)	30.3 (26.0)	33.5 (26.7)	63.1 (40.1)	48.0 (35.9)	33.6 (27.6)	34.2 (25.6)	
Age											
18-44 years	826 (66.4)	115.0 (67.9)	112.5 (74.5)	84.4 (60.8)	71.5 (65.6)	84.7 (67.5)	107.2 (66.2)	89.3 (66.8)	74.0 (60.8)	87.3 (65.4)	0.399
45 years or more	419 (33.6)	54.5 (32.1)	38.6 (25.5)	54.4 (39.2)	37.5 (34.4)	40.7 (32.5)	54.7 (33.8)	44.5 (33.2)	47.7 (39.2)	46.1 (34.6)	
Education level											
High school or less	558 (45.1)	32.7 (19.2)	39.0 (25.8)	86.2 (62.0)	48.9 (44.9)	70.1 (55.8)	88.7 (54.7)	69.8 (52.0)	64.3 (52.6)	65.6 (50.3)	< **0.001**
University	679 (54.9)	137.7 (80.8)	112.1 (74.2)	52.8 (38.0)	60.1 (55.1)	55.6 (44.2)	73.4 (45.3)	64.5 (48.0)	57.9 (47.4)	64.7 (49.7)	
Household income											
$60K or more	722 (63.1)	100.3 (62.5)	92.2 (64.8)	75.9 (61.0)	66.9 (66.3)	80.3 (68.8)	81.6 (57.3)	84.6 (66.1)	76.0 (65.1)	69.4 (58.5)	0.644
Less than $60K	423 (36.9)	60.2 (37.5)	50.0 (35.2)	48.4 (39.0)	34.0 (33.7)	36.4 (31.2)	60.9 (42.7)	43.4 (33.9)	40.8 (34.9)	49.2 (41.5)	
Parent											
No	777 (63.5)	107.2 (63.1)	96.6 (64.5)	88.1 (64.7)	66.4 (59.6)	71.1 (58.6)	104.1 (64.8)	89.5 (68.4)	81.5 (67.1)	80.0 (61.7)	0.739
Yes	447 (36.5)	62.6 (36.9)	53.2 (35.5)	48.1 (35.3)	45.0 (40.4)	50.3 (41.4)	56.5 (35.2)	41.4 (31.6)	39.9 (32.9)	49.6 (38.3)	
Health condition^b^											
No	788 (63.2)	97.5 (56.3)	82.9 (54.6)	86.5 (62.2)	69.5 (59.6)	85.0 (67.6)	103.6 (63.9)	95.6 (71.2)	83.8 (68.5)	92.1 (68.8)	**0.023**
Yes	459 (36.8)	75.7 (43.7)	69.0 (45.4)	52.5 (37.8)	47.0 (40.4)	40.7 (32.4)	58.4 (36.1)	38.6 (28.8)	38.4 (31.5)	41.7 (31.2)	
Province											
Alberta	159 (12.7)	20 (11.9)	15 (10.0)	26 (18.8)	11 (9.7)	13 (10.0)	21 (12.9)	17 (12.6)	17 (13.5)	20 (14.9)	0.549
British Columbia	161 (12.9)	17 (10.1)	20 (13.0)	24 (16.9)	17 (16.0)	12 (9.4)	31 (18.8)	15 (11.1)	13 (10.8)	12 (9.1)	
Manitoba	35 (2.8)	4 (2.5)	6 (3.8)	5 (2.6)	4 (3.6)	5 (3.7)	4 (2.4)	1 (1.0)	3 (2.6)	3 (2.0)	
New Brunswick	28 (2.2)	6 (3.4)	3 (2.2)	5 (3.3)	2 (1.4)	3 (2.3)	1 (0.8)	1 (0.8)	4 (3.4)	3 (2.3)	
Newfoundland and Labrador	12 (1.0)	3 (1.8)	1 (0.4)	1 (0.4)	0 (0.2)	1 (0.6)	2 (1.4)	2 (1.3)	1 (1.0)	2 (1.4)	
Nova Scotia	36 (2.9)	6 (3.3)	5 (3.5)	1 (0.7)	4 (3.5)	1 (0.9)	5 (2.9)	6 (4.6)	6 (4.6)	3 (2.2)	
Ontario	380 (30.4)	59 (34.9)	48 (31.7)	34 (24.3)	32 (29.7)	52 (41.6)	44 (26.9)	35 (25.9)	28 (23.0)	47 (35.3)	
Prince Edward Island	5 (0.4)	1 (0.3)	1 (1.0)	0 (0.3)	0 (0.0)	0 (0.0)	1 (0.5)	0 (0.0)	0 (0.3)	1 (0.6)	
Quebec	392 (31.4)	47 (27.8)	44 (29.4)	42 (30.4)	36 (33.4)	34 (26.8)	47 (29.1)	52 (39.0)	47 (38.2)	42 (31.4)	
Saskatchewan	40 (3.2)	6 (3.8)	8 (5.2)	2 (1.2)	3 (2.4)	6 (4.5)	7 (4.2)	5 (3.6)	3 (2.5)	1 (0.7)	

Bold denotes significance (*p* < 0.05); HCWs: healthcare workers; N_w_: number weighted. ^a^ Different survey period: Period 1 (April 9 – April 20, 2020); Period 2 (June 4 – June 17, 2020); Period 3 (October 29 – November 11, 2020); Period 4 (January 27 – February 7, 2021); Period 5 (March 11 – March 23, 2021); Period 6 (May 31 – June 14, 2021); Period 7 (September 10 – September 20, 2021); Period 8 (November 15 – December 3, 2021); Period 9 (January 20 – February 2, 2022). ^b^ Health condition at risk includes: any heart disease or history of heart attack or stroke, any chronic lung disease; active/current cancer; hypertension; diabetes; severe obesity; any autoimmune disease

## Results

### Participant characteristics

The sample consisted of 1615 HCWs over 9 assessment periods (see Table 1). The majority were female (66.0%), were aged 18-44 years (66.4%), had a university degree (54.9%,), had a household income ≥$60K or more (63.1%), did not have children (63.5%), and did not have a health condition (63.2%). See Table 2 for more details.

### COVID-19–related outcomes

Tables 3 and 4 present results for mental health and behavioural outcomes for the total sample and by sociodemographic variables (i.e., sex, age, education, income, parents, health status). In general, at least one out of five HCWs reported high levels of mental health problems in the last month, with the greatest proportion reporting feeling anxious (22.7%), isolated (22.3%), irritable (22.0%) and depressed (19.3%). Moreover, 39.0%, 26.4%, and 23.8% of HCWs reported decreasing their physical activity, eating less healthy diets, and drinking more alcohol, respectively, compared to pre-pandemic.

**Table 3:** Weighted prevalence of HCWs reporting mental health outcomes to a great extent by group, and tests for significance differences within groups

N_w_ (% category sample)	Anxious	D%	Depressed	D%	Isolated	D%	Felt irritable	D%
All	241 (22.7)		205 (19.3)		235 (22.3)		232 (22.0)	
Sex								
Female	192.2 (26.6)		156.1 (21.7)		169.6 (23.7)		178.3 (24.8)	
Male	44.2 (13.3)	-13.3	48.1 (14.4)	-7.3	60.8 (18.3)	-5.4	53.2 (16.0)	-8.8
N_w_ total	1055.3		1053.0		1048.1		1051.5	
Chi-square *p*-value	< **0.001**		**0.006**		**0.047**		**0.001**	
Age								
18-44 years	176.3 (25.1)		151.6 (21.7)		173.0 (24.7)		168.2 (24.1)	
45 years or more	63.5 (17.8)	-7.3	52.5 (14.7)	-7.0	60.6 (17.2)	-7.5	63.5 (17.8)	-6.3
N_w_ total	1058.7		1056.5		1051.5		1054.9	
Chi-square *p*-value	**0.007**		**0.007**		**0.005**		**0.021**	
Education level								
High school or less	113.4 (21.9)		109.6 (21.2)		130.7 (25.4)		115.1 (22.2)	
University	127.5 (23.9)	2.0	95.2 (17.9)	-3.3	104.3 (19.7)	-5.7	113.9 (21.5)	-0.7
N_w_ total	1050.3		1048.1		1043.12		1046.6	
Chi-square *p*-value	0.437		0.189		**0.027**		0.791	
Household income								
$60K or more	133.6 (21.8)		101.6 (16.5)		126.4 (20.8)		119.7 (19.5)	
Less than $60K	97.0 (27.0)	5.2	92.5 (26.0)	9.5	94.6 (26.4)	5.6	101.6 (28.6)	9.1
N_w_ total	972.9		970.4		965.5		968.9	
Chi-square *p*-value	0.067		< **0.001**		**0.045**		**0.001**	
Parent								
No	135.1 (20.4)		115.9 (17.6)		143.6 (21.9)		132.6 (20.2)	
Yes	99.3 (26.2)	5.8	80.5 (21.3)	3.7	81.2 (21.6)	-0.3	92.4 (24.2)	4.0
N_w_ total	1040.7		1038.5		1033.5		1037.3	
Chi-square *p*-value	**0.032**		0.143		0.904		0.130	
Health condition^a^								
No	134.5 (19.8)		109.0 (16.1)		139.8 (20.8)		139.6 (20.7)	
Yes	106.4 (27.8)	8.0	95.8 (25.0)	8.9	95.2 (24.9)	4.1	92.9 (24.2)	3.5
N_w_ total	1060.8		1058.6		1053.6		1057.1	
Chi-square *p*-value	**0.003**		< **0.001**		0.131		0.194	
Survey ^b^								
Time 1	-		-		-		-	
Time 2	27.7 (18.8)		14.5 (9.8)		16.9 (11.5)		23.9 (16.1)	
Time 3	33.5 (24.1)	5.3	31.0 (22.4)	12.6	24.1 (17.3)	5.8	27.7 (20.0)	3.9
Time 4	28.4 (26.3)	2.2	27.3 (25.5)	3.1	35.1 (32.6)	15.3	29.0 (27.0)	7.0
Time 5	32.4 (26.5)	0.2	32.7 (26.8)	1.3	37.4 (31.1)	-1.5	35.1 (28.9)	1.9
Time 6	43.0 (27.0)	0.5	29.2 (18.4)	-8.4	38.4 (24.7)	-6.4	31.3 (19.7)	-9.2
Time 7	27.3 (20.4)	-6.6	24.0 (18.0)	-0.4	35.0 (26.2)	1.5	26.1 (19.7)	0.0
Time 8	20.5 (17.1)	-3.3	21.3 (18.0)	0.0	14.8 (12.5)	-13.7	29.7 (25.5)	5.8
Time 9	28.2 (21.3)	4.2	24.7 (18.7)	0.7	33.3 (25.3)	12.8	29.6 (22.3)	-3.2
N_w_ total	1060.8		1058.6		1053.6		1057.1	
Chi-square *p*-value	0.367		**0.019**		< **0.001**		0.188	

Bold denotes significance (*p* < 0.05); *: no participant; D%: difference percentage; HCWs: healthcare workers; N_w_: number weighted: ^a^ Health condition at risk includes: any heart disease or history of heart attack or stroke, any chronic lung disease; active/current cancer; hypertension; diabetes; severe obesity; any autoimmune disease. ^b^ Different survey period: Period 1 (April 9 – April 20, 2020); Period 2 (June 4 – June 17, 2020); Period 3 (October 29 – November 11, 2020); Period 4 (January 27 – February 7, 2021); Period 5 (March 11 – March 23, 2021); Period 6 (May 31 – June 14, 2021); Period 7 (September 10 – September 20, 2021); Period 8 (November 15 – December 3, 2021); Period 9 (January 20 – February 2, 2022)

**Table 4:** Weighted prevalence of HCWs reporting COVID-19 impact on health behaviours by group, and tests for significance differences within groups

N_w_(% category sample)	Doing less physical activity	D%	Eating a less healthy diet	D%	Drinking more alcohol	D%	Smoking more cigarettes	D%	Vaping or using more e-cigarettes	D%	Using more recreational drugs	D%
All	359 (39.0)		243 (26.4)		219 (23.8)		110 (12.0)		78.3 (8.6)		125 (13.7)	
Sex												
Female	248.5 (39.5)		175.9 (28.1)		132.2 (21.1)		61.2 (9.8)		38.1 (6.1)		66.7 (10.6)	
Male	106.9 (37.3)	-2.2	63.1 (22.0)	-6.1	82.3 (28.8)	7.7	48.7 (17.1)	7.3	40.2 (14.2)	8.1	54.1 (19.0)	8.4
N_w_ total	915.9		914.0		912.2		911.1		907.7		910.9	
Chi-square *p*-value	**0.009**		< **0.001**		0.095		< **0.001**		< **0.001**		< **0.001**	
Age												
18-44 years	247.4 (41.6)		179.8 (30.2)		160.0 (26.9)		85.6 (14.4)		65.6 (11.1)		100.8 (17.0)	
45 years or more	111.4 (34.2)	-7.4	62.0 (19.3)	-10.9	58.2 (18.1)	-8.8	24.4 (7.6)	-6.8	12.7 (4.0)	-7.1	24.5 (7.6)	-9.4
N_w_ total	919.7		917.4		915.7		914.5		911.6		914.4	
Chi-square *p*-value	< **0.001**		< **0.001**		< **0.001**		**0.009**		< **0.001**		< **0.001**	
Education level												
High school or less	193.0 (39.9)		122.5 (25.4)		105.2 (21.7)		56.9 (11.8)		37.3 (7.8)		69.4 (14.4)	
University	162.4 (38.0)	-1.9	109.8 (25.8)	0.4	106.6 (25.2)	3.5	42.5 (10.0)	-1.8	33.6 (7.9)	0.1	55.9 (13.2)	-1.2
N_w_ total	910.9		909.0		907.3		906.2		902.8		906.0	
Chi-square *p*-value	**0.048**		0.492		0.684		0.229		0.564		0.310	
Household income												
$60K or more	195.4 (36.8)		141.8 (26.8)		137.8 (26.1)		55.6 (10.5)		43.1 (8.2)		62.9 (11.9)	
Less than $60K	131.7 (42.2)	5.4	82.2 (26.6)	-0.2	73.3 (23.7)	-2.4	50.9 (16.5)	6.0	34.0 (11.1)	2.9	60.3 (19.5)	7.6
N_w_ total	842.0		838.1		836.9		835.6		832.2		835.8	
Chi-square *p*-value	0.360		0.452		0.593		**0.003**		0.159		**0.002**	
Parental												
No	228.0 (39.8)		127.2 (22.2)		122.1 (21.3)		55.7 (9.8)		43.0 (7.6)		74.9 (13.1)	
Yes	125.0 (37.8)	-2.0	109.4 (33.3)	11.1	89.8 (27.5)	6.2	47.6 (14.5)	4.7	29.1 (8.9)	1.3	43.8 (13.4)	0.3
N_w_ total	903.0		901.1		900.5		898.9		895.1		898.7	
Chi-square *p*-value	0.079		**0.004**		**0.050**		0.083		0.107		0.719	
Health condition^a^												
No	240.5 (39.6)		146.6 (24.2)		127.9 (21.2)		51.0 (8.5)		43.5 (7.2)		73.4 (12.2)	
Yes	118.7 (37.9)	-1.7	96.3 (30.6)	6.4	90.8 (28.8)	7.6	58.9 (18.7)	10.2	34.8 (11.2)	5.0	52.0 (16.6)	4.4
N_w_ total	921.4		919.5		917.8		916.7		913.3		916.5	
Chi-square *p*-value	0.809		0.269		**0.019**		< **0.001**		**0.010**		**0.012**	
Survey^b^												
Time 1	-		-		-		-		-		-	
Time 2	-		-		-		-		-		-	
Time 3	46.8 (33.6)		33.6 (24.2)		31.1 (22.4)		15.4 (11.1)		8.5 (6.4)		16.7 (12.5)	
Time 4	47.4 (43.5)	9.9	33.1 (30.3)	6.1	28.9 (26.5)	4.1	11.8 (10.9)	-0.2	4.3 (4.0)	-2.4	7.3 (6.7)	-5.8
Time 5	47.0 (37.7)	-5.8	32.0 (25.6)	-4.7	34.1 (27.3)	0.8	15.1 (12.1)	1.2	14.3 (11.5)	7.5	22.1 (17.6)	10.9
Time 6	62.9 (38.9)	1.2	45.9 (28.6)	3.0	44.4 (27.7)	0.4	16.4 (10.1)	-2.0	12.2 (7.6)	-3.9	25.5 (15.8)	-1.8
Time 7	49.5 (37.0)	-1.9	34.7 (25.9)	-2.7	29.9 (22.4)	-5.3	21.1 (15.8)	5.7	11.6 (8.6)	1.0	14.2 (10.7)	-5.1
Time 8	47.3 (38.9)	1.9	24.9 (20.5)	-5.4	30.8 (25.5)	3.1	17.0 (14.5)	-1.3	16.6 (17.8)	9.2	19.2 (15.9)	5.2
Time 9	58.4 (44.4)	5.5	38.8 (29.6)	9.1	19.6 (15.0)	-10.5	13.2 (9.9)	-4.6	10.8 (8.2)	-9.6	20.2 (15.2)	-0.7
N_w_ total	921.4		919.5		917.8		916.7		913.3		916.5	
Chi-square *p*-value	0.149		0.106		0.137		0.707		< **0.001**		**0.019**	

Bold denotes significance (*p* < 0.05); *: no participant; D%: difference percentage; HCWs: healthcare workers; N_w_: number weighted. ^a^ Health condition at risk includes: any heart disease or history of heart attack or stroke, any chronic lung disease; active/current cancer; hypertension; diabetes; severe obesity; any autoimmune disease. ^b^ Period 1 (April 9 – April 20, 2020); Period 2 (June 4 – June 17, 2020); Period 3 (October 29 – November 11, 2020); Period 4 (January 27 – February 7, 2021); Period 5 (March 11 – March 23, 2021); Period 6 (May 31 – June 14, 2021); Period 7 (September 10 – September 20, 2021); Period 8 (November 15 – December 3, 2021); Period 9 (January 20 – February 2, 2022)

#### Sex

Female as compared with male HCWs reported feeling significantly more anxious (13.3% difference), depressed (7.3% difference), isolated (5.1% difference), and irritable (8.8 % difference). Female (vs. male) HCWs also reported engaging in significantly less physical activity (1.8% difference) and were eating less healthy diets (5.9% difference) compared to pre-pandemic. However, male (vs. female) HCWs reported smoking significantly more cigarettes (7.3% difference), using more e-cigarettes (8.1% difference), and using more recreational drugs (8.4% difference) compared to pre-pandemic.

#### Age

Younger (18-44 years of age) HCWs reported feeling significantly more anxious (7.3% difference), depressed (7.0% difference), isolated (7.5% difference), and irritable (6.3% difference) compared to HCWs 45 years of age or more. Finally, compared to older HCWs, younger HCWs also reported engaging in significantly less positive health behaviours since the pandemic started across all 6 behaviours assessed.

#### Education level

HCWs with a high school degree or less reported feeling significantly more isolated than those with a university degree (5.7% difference). HCWs with a high school degree or less were also significantly more likely to report engaging in less physical activity compared to pre-pandemic than those with a university degree (1.9% difference).

#### Household income

HCWs with household incomes below (vs. above) $60K reported feeling significantly more depressed (10.5% difference), isolated (5.6% difference), and irritable (9.1% difference). Also, compared to HCWs with incomes ≥$60K, HCWs with incomes less than $60K smoked more cigarettes (16.5% compared to 10.5%; *p*=0.003) and used more recreational drugs (19.5% compared to 11.9%; *p*=0.002) since the beginning of the pandemic.

#### Parental status

Compared to HCWs without children, those who were parents reported feeling significantly more anxious (5.8% difference). HCWs with (vs. without) children reported they were eating significantly less healthy diets (11.3% difference) and drinking more alcohol (6.2% difference) since the beginning of the pandemic.

#### Pre-existing physical health condition

HCWs with (vs. without) at least one medical health condition reported feeling significantly more anxious (8.0% difference), depressed (8.9% difference), and isolated (4.1% difference). HCWs with a medical health condition significantly, compared to those without, drank more alcohol (7.7% difference), smoked more cigarettes (9.8% difference), and used more e-cigarettes (4.0% difference) and recreational drugs (4.4% difference) since the beginning of the pandemic.

#### Changes over time

Over time, there were significant increases in HCWs who reported feeling more depressed, ranging from 9.8% (June 2020) to 32.6% (March 2021), and feeling more isolated, peaking at 26.8% in February 2021. For health behaviours, over time, there were significant increases in HCWs who were using e-cigarettes, ranging from 4.0% (January-February 2021) to 17.8% (November-December 2021), and using recreational drugs, ranging from 6.7% (January-February 2021) to 17.6% (March 2021). No other mental health or health behavioural outcomes had significant trend differences between the time points.

#### Predictors of mental health outcomes

Multivariable logistic regression analyses examining associations between mental health outcomes with all sociodemographic and health factors in one model across all surveys/time points are presented in Table 5. Female HCWs were 2.68 (95% CI 1.75 to 4.12) times more likely to report feeling more anxious, 1.63 (95% CI 1.02 to 2.59) times more depressed, and 1.61 (95% CI 1.08 to 2.40) times more irritable compared to male HCWs. Younger (18-44 years of age) HCWs were 1.79 (95% CI 1.18 to 2.71) times, 2.07 (95% CI 1.29 to 3.30) times, and 1.76 (95% CI 1.13 to 2.75) times more likely to feel more anxious, depressed, and isolated, respectively, compared to HCWs 45 years of age or more. HCWs with a high school degree or less were 1.53 (95% CI 1.06 to 2.22) times more likely to feel isolated than those with a university degree. HCWs with household incomes below (vs. above) $60K were 1.71 (95% CI 1.12 to 2.61) times more likely to feel depressed. HCWs without a health condition were less likely to report being anxious (OR = 0.50, 95% CI 0.35 to 0.72), depressed (OR = 0.48, 95% CI 0.32 to 0.71), and isolated (OR = 0.65, 95% CI 0.44 to 0.96) than those with at least one health condition. In November 2021, HCWs were 67% (OR = 0.33, 95% CI 0.16 to 0.68) more likely to feel isolated, compared to January 2022. Finally, in June 2020, HCWs were 61% (OR = 0.39, 95% CI 0.18 to 0.83) and 64% (OR = 0.36, 95% CI 0.18 to 0.72) more likely to feel depressed and isolated, respectively, compared to January 2022. These results are, by and large, consistent with the results from the bivariate analyses presented above, with few exceptions (i.e., being a parent was no longer significantly associated with feeling anxious, sex was no longer associated with feeling isolated, and health conditions were now significantly associated with feeling isolated).

**Table 5:** Multivariate^a^ associations between sociodemographic characteristics and health conditions with mental health outcomes during COVID-19 among HCWs

COVID-19-impact	Anxious			Depressed			Isolated			Felt irritable		
Variable	OR^c^	95% confidence interval		OR	95% confidence interval		OR	95% confidence interval		OR	95% confidence interval	
		Lower	Upper		Lower	Upper		Lower	Upper		Lower	Upper
Sex												
Male	1			1			1			1		
Female	**2.68**	**1.75**	**4.12**	**1.63**	**1.02**	**2.59**	1.34	0.87	2.07	**1.61**	**1.08**	**2.40**
Age												
45 years and more	1			1			1			1		
18-44 years	**1.79**	**1.18**	**2.71**	**2.07**	**1.29**	**3.30**	**1.76**	**1.13**	**2.75**	1.54	1.00	2.38
Education												
University	1			1			1			1		
High school or less	0.94	0.66	1.35	1.19	0.82	1.73	**1.53**	**1.06**	**2.22**	1.07	0.75	1.54
Income												
$60K or more	1			1			1			1		
Less than $60K	1.30	0.88	1.93	**1.71**	**1.12**	**2.61**	1.15	0.76	1.75	**1.65**	**1.10**	**2.48**
Parent												
Yes	1			1			1			1		
No	0.80	0.55	1.16	0.86	0.57	1.31	1.10	0.73	1.65	0.90	0.61	1.33
Health condition^b^												
Yes	1			1			1			1		
No	**0.50**	**0.35**	**0.72**	**0.48**	**0.32**	**0.71**	**0.65**	**0.44**	**0.96**	0.75	0.51	1.10
Survey^d^												
Time 1	-*	-	-	-	-	-	-	-	-	-	-	-
Time 2	0.73	0.37	1.43	**0.39**	**0.18**	**0.83**	**0.36**	**0.18**	**0.72**	0.72	0.37	1.43
Time 3	1.29	0.62	2.70	1.30	0.59	2.84	0.51	0.23	1.15	1.04	0.47	2.30
Time 4	1.02	0.48	2.17	1.17	0.56	2.49	1.28	0.63	2.63	1.24	0.58	2.63
Time 5	1.40	0.70	2.77	1.40	0.67	2.92	1.10	0.55	2.20	1.72	0.87	3.39
Time 6	1.37	0.69	2.74	0.97	0.47	2.01	0.82	0.41	1.66	1.08	0.54	2.18
Time 7	1.07	0.52	2.21	1.04	0.48	2.26	1.08	0.54	2.15	1.09	0.51	2.33
Time 8	0.65	0.32	1.32	0.75	0.36	1.55	**0.33**	**0.16**	**0.68**	1.29	0.59	2.78
Time 9	1			1			1			1		

Bold denotes significance (*p* < 0.05); *: survey at Time 1 did not include reported outcome measures. ^a^ Multivariate logistic regression was conducted to assess the relationships between sociodemographic and mental health outcomes; analysis was done on 1261, 1257, 1252, 1254 individuals due to missing values in either response or explanatory variables, respectively. ^b^ Health condition at risk includes: any heart disease or history of heart attack or stroke, any chronic lung disease; active/current cancer; hypertension; diabetes; severe obesity; any autoimmune disease. ^c^ OR—odds ratio. ^d^ Period 1 (April 9 – April 20, 2020); Period 2 (June 4 – June 17, 2020); Period 3 (October 29 – November 11, 2020); Period 4 (January 27 – February 7, 2021); Period 5 (March 11 – March 23, 2021); Period 6 (May 31 – June 14, 2021); Period 7 (September 10 – September 20, 2021); Period 8 (November 15 – December 3, 2021); Period 9 (January 20 – February 2, 2022)

#### Predictors of health behaviour outcomes

Multivariable logistic regression analyses examining associations between health behaviour change with sociodemographic and health factors across all surveys/time points are presented in the Table 6. Since the beginning of the pandemic, female HCWs were 1.54 (95% CI 1.02 to 2.31) times more likely to eat less healthy diets compared with male HCWs, but males were 63% (OR = 0.37, 95% CI 0.19 to 0.72) and 51% (OR = 0.49, 95% CI 0.29 to 0.82) more likely to use more e-cigarettes and recreational drugs, respectively. Younger (vs. older) HCWs were 1.70 (95% CI 1.06 to 2.71) times and 2.41 (95% CI 1.22 to 4.77) times more likely to eat a less healthy diet and used more recreational drugs, respectively, since the beginning of the pandemic. HCWs with (vs. those without) children were 38% (OR = 0.62, 95% CI 0.41 to 0.94) more likely to report they were eating significantly less healthy diets since the beginning of the pandemic. HCWs without a medical health condition (vs. those with at least one) were 46% (OR = 0.54, 95% CI 0.29 to 0.99) more likely to smoke more cigarettes since the beginning of the pandemic. HCWs were 1.92 (95% CI 1.02 to 3.62) times more likely to drink more alcohol in June 2021 than in January 2022. Also, HCWs were 2.76 (95% CI 1.07 to 7.13) and 3.20 (95% CI 1.25 to 8.21) times more likely to smoke cigarettes during September and November 2021, respectively, than in January 2022. Finally, in June 2020, HCWs were 65% (OR = 0.35, 95% CI 0.15 to 0.82) more likely to use recreational drugs than in January 2022.

**Table 6:** Multivariate^a^ associations between sociodemographic characteristics and health conditions with health behaviours outcomes compared to before the COVID-19 pandemic among HCWs

Health behaviour^a^	Doing less physical activity†				Eating a less healthy diet†				Drinking more alcohol†			
Variable	OR^c^	95% confidence interval			OR	95% confidence interval			OR	95% confidence interval		
		Lower	Upper			Lower	Upper			Lower	Upper	
Sex												
Male	1				1				1			
Female	1.13	0.78	1.62		**1.54**	**1.02**	**2.31**		0.71	0.47	1.07	
Age												
45 years and more	1				1				1			
18-44 years	1.37	0.92	2.04		**1.70**	**1.06**	**2.71**		1.54	0.97	2.46	
Education												
University	1				1				1			
High school or less	1.19	0.85	1.68		1.18	0.81	1.71		0.94	0.64	1.38	
Income												
$60K or more	1				1				1			
Less than $60K	1.28	0.87	1.89		0.93	0.69	1.44		0.90	0.57	1.41	
Parent												
Yes	1				1				1			
No	1.04	0.72	1.50		**0.62**	**0.41**	**0.94**		0.84	0.56	1.26	
Health condition^b^												
Yes	1				1				1			
No	0.91	0.63	1.32		0.70	0.47	1.06		0.71	0.47	1.06	
Survey^d^												
Time 1	-*	-	-		-	-	-		-	-	-	
Time 2	-*	-	-		-	-	-		-	-	-	
Time 3	0.52	0.27	1.02		0.87	0.40	1.89		1.62	0.81	3.24	
Time 4	0.84	0.45	1.57		0.93	0.46	1.86		1.61	0.82	3.16	
Time 5	0.70	0.37	1.32		0.95	0.45	2.00		1.81	0.94	3.50	
Time 6	0.67	0.37	1.23		0.86	0.43	1.72		1.92	1.02	3.62	
Time 7	0.65	0.34	1.22		0.87	0.41	1.85		1.35	0.68	2.67	
Time 8	0.74	0.40	1.39		0.69	0.33	1.43		1.85	0.96	3.58	
Time 9	1				1				1			
Health behaviour^a^	Smoking more cigarettes‡				Vaping or using more e-cigarettes‡				Using more recreational drugs‡			
Variable	OR^c^	95% confidence interval			OR	95% confidence interval			OR	95% confidence interval		
		Lower	Upper			Lower	Upper			Lower	Upper	
Sex												
Female	1				1				1			
Male	0.56	0.31	1.01		**0.37**	**0.19**	**0.72**		**0.49**	**0.29**	**0.82**	
Age												
18-44 years	1				1				1			
45 years or more	1.72	0.85	3.49		2.38	0.89	6.32		**2.41**	**1.22**	**4.77**	
Education level												
High school or less	1				1				1			
University	1.20	0.70	2.06		1.14	0.61	2.10		1.19	0.73	1.93	
Income												
$60K or more	1				1				1			
Less than $60K	1.83	0.97	3.44		1.45	0.69	3.08		1.73	0.98	3.05	
Parent												
No	1				1				1			
Yes	0.70	0.40	1.25		1.02	0.52	2.00		1.09	0.65	1.82	
Health condition^b^												
No	1				1				1			
Yes	**0.54**	**0.29**	**0.99**		0.78	0.40	1.53		0.59	0.34	1.02	
Survey^d^												
Time 1	-*	-	-		-	-	-		-	-	-	
Time 2	-*	-	-		-	-	-		-	-	-	
Time 3	1.94	0.67	5.62		0.96	0.24	3.92		0.61	0.22	1.65	
Time 4	1.46	0.45	4.76		0.74	0.22	2.45		**0.35**	**0.15**	**0.82**	
Time 5	1.84	0.65	5.25		1.63	0.44	5.97		0.89	0.35	2.26	
Time 6	1.78	0.64	4.95		1.17	0.33	4.14		0.70	0.29	1.67	
Time 7	**2.76**	**1.07**	**7.13**		0.91	0.25	3.32		0.57	0.22	1.45	
Time 8	**3.20**	**1.25**	**8.21**		2.85	0.84	9.67		0.89	0.36	2.21	
Time 9	1				1				1			

Bold denotes significance (*p* < 0.05); *: surveys at Time 1 and Time 2 did not include reported outcome measures. ^a^ Multivariate logistic regression was conducted to assess the relationships between sociodemographic and health behaviours; analysis was done on 1065†, 1095†, 1121†, 1120‡, 1115‡, and 1119‡ individuals respectively due to missing values in either response or explanatory variables. ^b^ Health condition at risk includes: any heart disease or history of heart attack or stroke, any chronic lung disease; active/current cancer; hypertension; diabetes; severe obesity; any autoimmune disease. ^c^ OR—odds ratio. ^d^ Period 1 (April 9 – April 20, 2020); Period 2 (June 4 – June 17, 2020); Period 3 (October 29 – November 11, 2020); Period 4 (January 27 – February 7, 2021); Period 5 (March 11 – March 23, 2021); Period 6 (May 31 – June 14, 2021); Period 7 (September 10 – September 20, 2021); Period 8 (November 15 – December 3, 2021); Period 9 (January 20 – February 2, 2022)

## Discussion

The results of this study including multiple surveys over the first two years of the pandemic demonstrated significant impacts of COVID-19 on Canadian HCWs’ mental health outcomes and health behaviours, which varied by sociodemographic factors and health status. Between April 9, 2020 and February 2, 2022, ~20-23% of HCWs reported being anxious, irritable, isolated, or depressed to a great extent. Furthermore, nearly 40% of HCWs reported engaging in less physical activity during the first two years since the pandemic started, and approximately one quarter reported eating less healthy diets and drinking alcohol more. A peak is observed for mental health outcomes and poor health behaviours between March and June 2021.

While there was some variability across mental health and health behaviour outcomes, in general, HCWs who identified as female, were younger, had annual household incomes below $60K, or were living with a physical health condition reported worse mental health throughout the pandemic than their counterparts. Similarly, younger HCWs, those with a lower household income, and those who were living with a physical health condition reported consistently worse health behaviours than their counterparts. Finally, whereas female (as compared with male) HCWs reported reductions in healthy eating and physical activity since the start of the pandemic, male HCWs reported greater use of recreational drugs and cigarettes (combustible or e-cigarettes). The totality of these findings speaks to significant, wide-ranging mental health and behavioural impacts of the pandemic on HCWs that varied by sociodemographic and health status.

Mental health results for the full sample of HCWs in the present study are consistent with, though lower than, previous national reports which showed that 36% and 26% of Canadian HCWs reported significant depression or anxiety, respectively (Canadian Medical Association, 2018; Stelnicki & Carleton, 2021). The iCARE study, from which these data were retrieved, had slightly lower proportions of HCWs reporting mental health issues, perhaps attributable to the fact that the current study utilized single items to assess each mental health outcome. Previous Canadian and international studies have also shown significant mental health effects resulting from the pandemic that varied by sociodemographic factors (Pierce et al., 2020) and health status (Deslauriers et al., 2022), but few have examined whether such differences existed in HCWs. Our study revealed that the pandemic disproportionately impacted similar groups as in the general population, namely females (Jenkins et al., 2021), younger people (El-Gabalawy & Sommer, 2021), individuals with children (Gadermann et al., 2021), lower socioeconomic status individuals (Miconi et al., 2021; Raina et al., 2021), and those experiencing health problems (Deslauriers et al., 2022). These results highlight the need for wide-scale and targeted wellness and behavioural intervention programming within healthcare systems across the country (Yang et al., 2021).

### Future directions

Many interventions for HCWs focus on mental health education, access to psychological support, or a multidisciplinary approach (i.e., peer support, health consultant; Buselli et al., 2021; Robins-Browne et al., 2022), but few interventions address the mental health of HCWs through behaviour change initiatives targeting specific behavioural outcomes (e.g., physical activity, alcohol consumption). In comparison with before the COVID-19 pandemic, the present study revealed that overall, HCWs have changed their health behaviours, with more than a third being less physically active (38%) and more than a quarter eating a less healthy diet (26%). Also, alcohol consumption, cigarette or e-cigarette use, and recreational drug use were more notable in the present study. It would seem that those most at risk for engaging in more unhealthy behaviours were HCWs who were male, were 18-44 years of age, had a household income under $60K, or had a health condition, a trend that was similarly observed very early on in the pandemic globally (Arora & Grey, 2020) and for the general Canadian population (Zajacova et al., 2020).

### Limitations of the study

Our sample of HCWs was drawn from the larger iCARE study and did not intentionally or actively target recruitment of HCWs. A survey with HCWs as the target population could have potentially increased the sample size or accuracy of the data. However, the proportion of HCWs in our samples represented 5.9% of the full iCARE sample, which is consistent with the proportional representation of Canadians who identify as HCWs (5.5%). Additionally, we do not have the specific profession of HCWs (e.g., registered nurses, physicians, administrative staffs), limiting the interpretation and generalizability of the results knowing that COVID-19 may have had different impacts depending on profession type (Statistics Canada, 2021). A second limitation is that the mental health and behavioural questions were single items developed for their face/ecological validity (as often done in broad epidemiological research) and were not validated or standardized questionnaires. Including comprehensive questionnaires for mental health-related symptoms could be considered for future iterations, and greater efforts to validate single item questions and to ensure their reliability are needed. While representative sampling has strengths (e.g., proportion of the population by sociodemographic characteristics, random-digit sampling), it is not without selection bias. As previously shown (Joyal-Desmarais et al., 2022), the samples from the Leger panel were not fully representative of the Canadian population (e.g., age, language, education). Also, due to the sampling methods of the Leger Opinion polling firm, it is not possible to know whether a respondent has participated in and contributed to several data collection times. Finally, we report the results of a series of surveys which were sent during different phases of the COVID-19 pandemic and to different participants (cohorts) at each time point. As this is cross-sectional data, it is not possible to track individual changes over time, and these kinds of analyses would add to the current study.

## Conclusion

The current study found that HCWs—especially those who identified as female, were younger, had one or more health conditions, or had an income of less than $60K—experienced worse mental health outcomes and engaged in poorer health behaviours during the first 2 years of the pandemic. Although this study does not show the direct links of COVID-19–related policies instituted in healthcare settings and mental health or health behaviours of HCWs, it should not be ignored that these outcomes need to be a primary focal target of Canadian and provincial healthcare ministries.

## Contributions to knowledge

What does this study add to existing knowledge?

- Our findings add to the literature around the mental health and health behaviours of healthcare workers during the COVID-19 pandemic.
- Results from our study indicate that healthcare workers who were female, younger, living with one or more health conditions, or having a household income of less than $60K were the most impacted.

What are the key implications for public health interventions, practice or policy?

- These results direct attention to the need to develop behavioural intervention strategies that directly target the healthcare workers most disproportionately impacted by the pandemic.
- Canadian health administrations need to address these growing disparities through institutional policies and wellness programming.

## Supplementary Information

ESM 1 (DOCX 28 kb)

ESM 2 (DOCX 28 kb)

## Data Availability

Surveys information, protocol and project content can be found here: https://osf.io/nswcm/
