# Supplementary material for: Canadian healthcare workers’ mental health and health behaviours during the COVID-19 pandemic: results from nine representative samples between April 2020 and February 2022
Source: Can J Public Health. 2023 Aug 7;114(5):823–39. doi: 10.17269/s41997-023-00807-z (PMC10485207; doi:10.17269/s41997-023-00807-z)
Supplement: Supplementary file 2 — (DOCX 28 kb) [file 41997_2023_807_MOESM2_ESM.docx]

**Supplementary Table 2. Questions that are used from the iCARE Canadian representative sample survey**

| **Code** | **Questions** | **Possible responses** | **Variable modification for analyse** |
| --- | --- | --- | --- |
| **HCW identification** | Are you a healthcare worker? | 1, No \| 2, Yes \| 99, I don't know/I prefer not to answer | “Yes” for inclusion |
|  |  |  |  |
| **Sex** | How would you describe your sex? | 1. Male \| 2, Female\| 3, Other \| 9, I prefer not to answer | “Male”  vs.  “Female” |
| **Age** | What is your age? | 0, Under 18 \| 1, Between 18 and 24 \| 2, Between 25 and 34 \| 3, Between 35 and 44 \| 4, Between 45 and 54 \| 5, Between 55 and 64 \|6, Between 65 and 74 \| 7, 75 or older \| 9, I prefer not to answer | 18-24 years  25-34 years  35-44 years  vs.  45-54 years  55-64 years  65-74 years  75 years or + |
| **Education level** | What is your highest level of completed education? | 1, Primary or elementary school or less \| 2, Secondary or high school \| 3, College or University degree \| 4, Graduate or Postgraduate degree \| 5, I have never been to school \| 9, I don’t know or I prefer not to answer | “Primary or elementary school or less”, “Secondary or high school”  vs.  “College or University degree”, “Graduate or Postgraduate degree” |
| **Household income** | Which of the following categories best reflects the total INCOME before tax of all members of your household for the year 2019? | 1, 19 999$ and less \| 2, From $ 20 000 to $ 39 999 \| 3, From $ 40 000 to $ 59 999 \| 4, From $ 60 000 to $ 79 999 \| 5, From $ 80 000 to $ 99 999 \| 6, From $ 100 000 to $ 149 999 \| 7, $ 150 000 and more \| 99, I'd rather not answer | “19 999$ and less”, “From $ 20 000 to $ 39 999”, “From $ 40 000 to $ 59 999”  vs.  “From $ 60 000 to $ 79 999”, “From $ 80 000 to $ 99 999”, “From $ 100 000 to $ 149 999”, “$ 150 000 and more” |
| **Parental status** | Are you a parent of any of these children? | 1, No \| 2, Yes \| 99, I don't know/I prefer not to answer | “Yes”  vs.  “No” |
| **Health condition** | Question for (1) Any heart disease or history of heart attack or stroke, (2) Any chronic lung disease, (3) Active/current cancer, (4) Hypertension, (5) Diabetes, (6) Severe obesity and (7) Any autoimmune disease.  “Medical condition”… To your knowledge, has a doctor or healthcare professional told you that you have any of the following health conditions? | 1, Yes \| 2, No \| 3, I don't know/I prefer not to answer | “Yes”  vs.  “No” |
|  |  |  |  |
| **Psychological outcome 1** | COVID-19 has affected several aspects of people’s lives. Please rate the extent to which COVID-19 has impacted the following aspects of your life over the last month:  Because of COVID-19…I have felt nervous, anxious, or worried | 1, To a Great Extent \| 2, Somewhat \| 3, Very Little \| 4, Not at All \| 97, Not applicable \| 99, I don’t know or I prefer not to answer \| | “To a Great Extent”  vs.  all other choices |
| **Psychological outcome 2** | COVID-19 has affected several aspects of people’s lives. Please rate the extent to which COVID-19 has impacted the following aspects of your life over the last month:  Because of COVID-19…I have felt sad, depressed, or hopeless | 1, To a Great Extent \| 2, Somewhat \| 3, Very Little \| 4, Not at All \| 97, Not applicable \| 99, I don’t know or I prefer not to answer \| | “To a Great Extent”  vs.  all other choices |
| **Psychological outcome 3** | COVID-19 has affected several aspects of people’s lives. Please rate the extent to which COVID-19 has impacted the following aspects of your life over the last month:  Because of COVID-19…I have felt lonely and isolated | 1, To a Great Extent \| 2, Somewhat \| 3, Very Little \| 4, Not at All \| 97, Not applicable \| 99, I don’t know or I prefer not to answer \| | “To a Great Extent”  vs.  all other choices |
| **Psychological outcome 4** | COVID-19 has affected several aspects of people’s lives. Please rate the extent to which COVID-19 has impacted the following aspects of your life over the last month:  Because of COVID-19…I have felt irritable, frustrated or angry | 1, To a Great Extent \| 2, Somewhat \| 3, Very Little \| 4, Not at All \| 97, Not applicable \| 99, I don’t know or I prefer not to answer \| | “To a Great Extent”  vs.  all other choices |
| **Health behaviour 1** | In general, how have the following behaviours changed since the start of COVID-19?  Doing physical activity | 1, I do this a lot more\| 2, I do this more \| 3, I do this as much as before \| 4, I do this less \| 5, I do this a lot less \| 6, I don’t do this \| 7, I don’t know/I prefer not to answer | “I do this a lot less”, “I do this less”  vs.  all other choices |
| **Health behaviour 2** | In general, how have the following behaviours changed since the start of COVID-19?  Eating a healthy diet | 1, I do this a lot more\| 2, I do this more \| 3, I do this as much as before \| 4, I do this less \| 5, I do this a lot less \| 6, I don’t do this \| 7, I don’t know/I prefer not to answer | “I do this a lot less”, “I do this less”  vs.  all other choices |
| **Health behaviour 3** | In general, how have the following behaviours changed since the start of COVID-19?  Drinking alcohol | 1, I do this a lot more \| 2, I do this more \| 3, I do this as much as before \| 4, I do this less \| 5, I do this a lot less \| 6, I don’t do this \| 7, I don’t know/I prefer not to answer | “I do this a lot more”, “I do this more”  vs.  all other choices |
| **Health behaviour 4** | In general, how have the following behaviours changed since the start of COVID-19?  Smoking cigarettes | 1, I do this a lot more \| 2, I do this more \| 3, I do this as much as before \| 4, I do this less \| 5, I do this a lot less \| 6, I don’t do this \| 7, I don’t know/I prefer not to answer | “I do this a lot more”, “I do this more”  vs.  all other choices |
| **Health behaviour 5** | In general, how have the following behaviours changed since the start of COVID-19?  Vaping or using electronic cigarettes | 1, I do this a lot more \| 2, I do this more \| 3, I do this as much as before \| 4, I do this less\| 5, I do this a lot less \| 6, I don’t do this \| 7, I don’t know/I prefer not to answer | “I do this a lot more”, “I do this more”  vs.  all other choices |
| **Health behaviour 6** | In general, how have the following behaviours changed since the start of COVID-19?  Using recreational drugs | 1, I do this a lot more \| 2, I do this more \| 3, I do this as much as before \| 4, I do this less \| 5, I do this a lot less \| 6, I don’t do this \| 7, I don’t know/I prefer not to answer | “I do this a lot more”, “I do this more”  vs.  all other choices |
